# Artificial Intelligence-Enabled Computer Vision for Food Inspection: From Validated Measurements to Process Control

**DOI:** 10.3390/foods15203591

**Published:** 2026-10-10

**Authors:** Na Xu, Yi Lu

**Affiliations:** 1 Department of Translational Genomics, Faculty of Health, Medicine & Life Sciences, GROW Institute for Oncology and Reproduction, Maastricht University, 6200 MD Maastricht, The Netherlands; 2 Department of Human Genetics, Radboud University Medical Center, 6525 GA Nijmegen, The Netherlands; luyi920127@163.com

**Keywords:** computer vision, artificial intelligence, food quality and safety, external validation, process control

## Abstract

Artificial intelligence-enabled computer vision can increase the frequency and spatial coverage of food inspection, but high predictive accuracy does not establish reliable production decisions. This critical narrative review examines the evidence connecting image measurements with grading, rejection, and process control. Representative studies across produce, grains and nuts, meat and seafood, microbiology, and packaging are compared by sensing modality, reference endpoint, validation unit, and operational action. The synthesis distinguishes visible quality from specific hazards, prepared defects from natural variation, and image-level performance from verified product outcomes. An author-proposed evidence map organizes analytical feasibility, independent-sample testing, operational challenges, external transfer, and prospective impact as partly independent dimensions. It is unvalidated and is not a readiness score or regulatory standard. Task-dependent reporting considerations address uncertainty, prevalence, false rejection, and abstention. Safety-related uses require separate hazard-specific validation, sampling, and acceptance criteria. Process integration additionally requires acquisition-to-actuation testing, accepted-product verification, and controlled updates. The purposive evidence base cannot establish field-wide effectiveness or rank modalities. Research priorities include traceable datasets, mechanistic challenges, and prospective intervention studies. Progress toward closed-loop control should be judged by stable measurements and demonstrated effects on defined product and process outcomes within a stated operating domain.

## 1. Introduction

Food inspection must distinguish acceptable biological variation from changes that compromise quality, authenticity, or safety. Color, shape, surface moisture, tissue structure, and composition vary with cultivar, raw-material source, processing, and storage. The same visible change can therefore have different meanings in different products: browning may indicate desirable baking, mechanical damage, enzymatic oxidation, or deterioration. Conversely, a product with an acceptable appearance may contain an internal defect or a hazard without a visible signature. Inspection technologies must be evaluated against this food-specific relationship between the measured signal, the reference endpoint, and the decision that follows.

Computer vision converts images into measurements, classifications, or localized detections. In food applications, it encompasses conventional image processing and artificial intelligence (AI) applied to red–green–blue (RGB), multispectral, hyperspectral, fluorescence, thermal, X-ray, computed-tomography (CT), and three-dimensional (3D) data. Foundational reviews established its contribution to automated inspection and produce grading [1,2]. Subsequent reviews describe the expansion toward deep learning, spectral analysis, and integrated food-processing systems [3,4,5,6]. The resulting literature offers many technically promising solutions, but an algorithmic improvement and an improvement in food control are different outcomes.

A high test-set accuracy does not establish that a model will work on new production. Images, spectral pixels, or video frames can be numerous while independent fruits, fillets, packages, or batches remain few. When related observations cross the training–test boundary, evaluation can reward recognition of acquisition context rather than generalization. Even without leakage, performance may change with season, supplier, instrument, sanitation state, or packaging atmosphere. In addition, a statistically useful model may be operationally unsuitable because its response is late, its thresholds reject too much acceptable food, or its predictions cannot be linked to the correct item.

Safety claims require particular restraint. Detecting discoloration, predicting a freshness indicator, identifying a cultured microcolony, and demonstrating the absence of a pathogen are distinct analytical claims. Likewise, observing a seal irregularity does not establish package leakage, and locating a foreign object does not prove that it was removed. These distinctions determine which reference methods, challenge samples, and verification activities are necessary. A food-vision system should be understood as one component of an established control strategy, with a clearly bounded intended use.

This review examines the evidence needed to move AI-enabled food inspection from image recognition to reliable production decisions. Its central question is: under what measurement, validation, and operating conditions can a vision output justify grading, holding, rejecting, or adjusting a food process? Representative studies across produce, grains and nuts, meat and seafood, microbiological testing, and packaging are compared through this question. The contribution is a critical synthesis connecting food-state observability, independent validation, decision thresholds, acquisition-to-action performance, and lifecycle verification. A proposed evidence map, organized using five evidence-dimension labels, and task-dependent reporting tables organize the synthesis; they are not a consensus standard or a validated readiness instrument.

## 2. Review Approach and Evidence Boundaries

This is a critical narrative review, with a focused literature update completed on 5 September 2026. The evidence base was assembled from the cited food-imaging literature and supplemented by targeted title, digital object identifier (DOI), and keyword searches of publisher records and PubMed/PubMed Central. Representative keyword combinations included “computer vision” with “food inspection” and “external validation,” and “food,” “hyperspectral,” and “data leakage.” Targeted follow-up searches addressed packaging, poultry foreign materials, mycotoxins, and microbial imaging. Crossref records and publisher or conference records were used to check bibliographic information. The emphasis is on studies published from 2015 onward, with earlier references retained for methodological context. Targeted reference-status checks and methodological additions for this revision were completed on 27 September 2026; this revision check is not a new systematic search.

Studies were selected for their relevance to an inspection decision and their ability to illustrate measurement capability, validation design, or process integration. Eligible application examples concerned foods intended for human consumption, food packages, or microbial testing relevant to foods. General machine-learning papers provide methodological support but are not counted as evidence of food-industry performance. Dietary-image recognition, preharvest crop monitoring without an inspection connection, feed-only applications, and non-imaging prediction studies are outside the application synthesis.

The comparison considers the food matrix, imaging modality, reference endpoint, prediction unit, reported metric, evaluation setting, and operational evidence. Particular attention is paid to physical-sample independence, external testing, metric denominators, and the distinction between inference speed and complete system throughput.

To reduce selective interpretation, the revision compares the selected examples using the same extraction fields and retains unfavorable findings, live-test performance reductions, residual misses, and uncertain sample independence alongside favorable results. Application reports are interpreted against their stated reference endpoint and operating environment, and methodological papers are separated from empirical food-performance evidence. These safeguards improve transparency but cannot demonstrate unbiased selection.

The literature was assembled purposively, without a registered protocol, exhaustive database strategy, duplicate independent screening, or a formal validated risk-of-bias instrument. The search examples above describe the approach rather than a reproducible systematic-review search. Table S1 provides a structured qualitative appraisal of the selected empirical examples, including missing or unresolved information; it is not an exhaustive evidence inventory. Accessible reports and frequently studied RGB/HSI applications and commodities may be overrepresented, and publication and language/accessibility biases cannot be excluded. Consequently, no pooled performance, prevalence of successful deployment, or ranking of modalities is inferred. Narrative-review quality principles support transparent objectives, source selection, reasoning, and endpoint reporting [7], while PRISMA addresses systematic reviews [8].

## 3. Connecting Food State, Image Information, and Action

### 3.1. The Measurement-to-Control Chain

**Figure 1:**
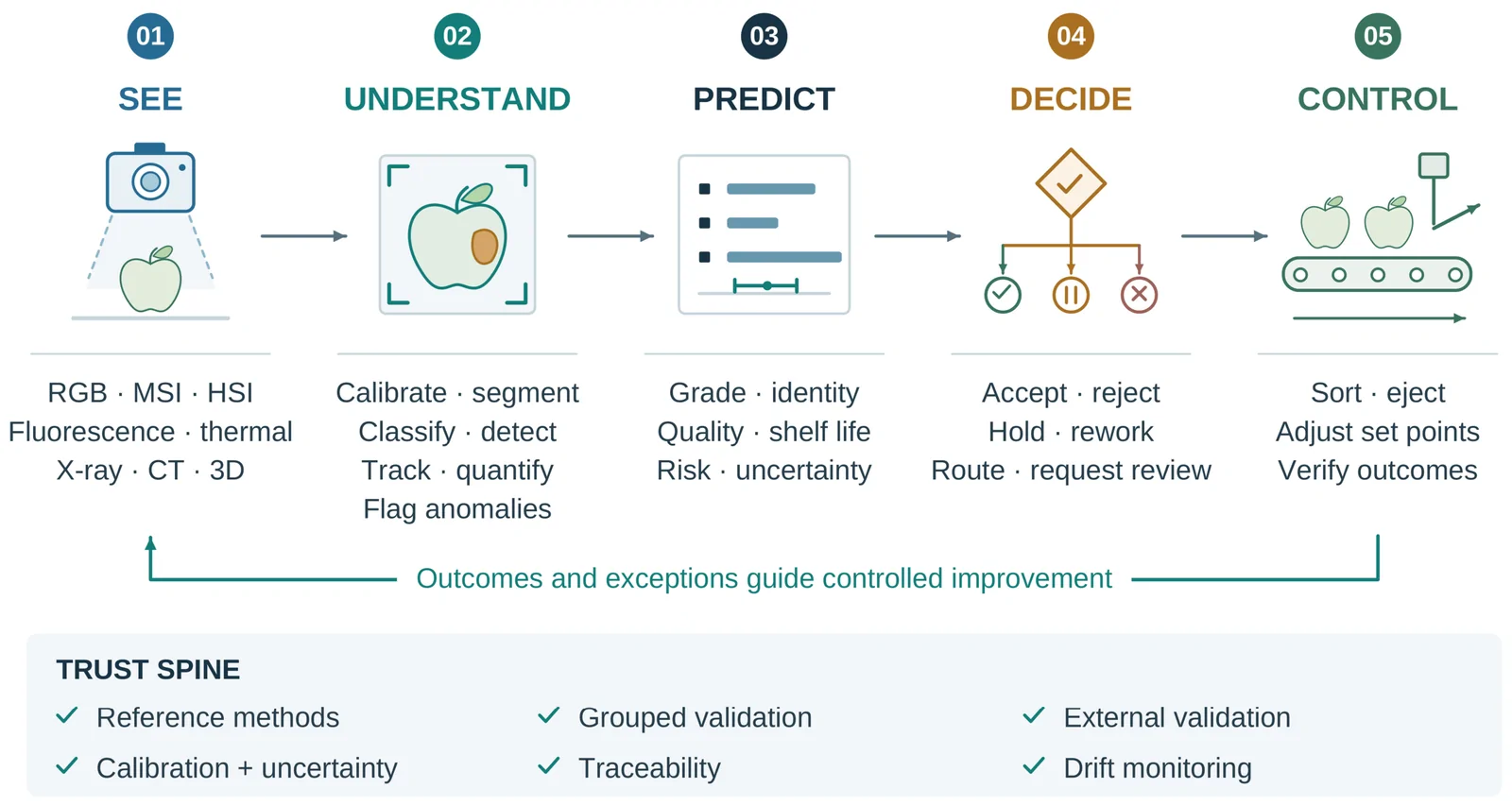
Proposed measurement-to-control chain for food inspection. Seeing, understanding, predicting, deciding, and controlling link acquisition to verified action. The return path represents outcome information used for controlled improvement. The lower panel labeled “TRUST SPINE” denotes supporting validation and traceability activities, not a certification standard. RGB, red–green–blue; MSI, multispectral imaging; HSI, hyperspectral imaging; CT, computed tomography; 3D, three-dimensional.

Figure 1 organizes an inspection system into seeing, understanding, predicting, deciding, and controlling. Acquisition determines what information is available; calibration, segmentation, and feature extraction determine how it is represented; prediction relates that information to a defined endpoint. A separate decision rule then assigns an action. The control stage includes implementation and verification of that action. Error at an earlier stage propagates forward: a better classifier cannot recover an occluded contaminant, correct an inappropriate chemical reference, or repair a mismatch between the detected item and the ejector.

The intended use should therefore precede model selection. A useful specification identifies the product and operating range, the defect or attribute, its reference definition, the spatial or analytical resolution required, and the allowed response. For example, locating a surface bruise to guide trimming requires an area estimate and an acceptable trimming margin. Predicting soluble solids for grade assignment requires error limits around grade boundaries. Detecting foreign material requires object-level sensitivity under specified sizes, materials, and positions, followed by evidence of correct removal. These tasks may use related architectures but require different acceptance criteria.

Three operating modes should remain distinct. Decision support presents measurements to an operator. Automated sorting applies a fixed rule to route individual products. Closed-loop process control uses measured outcomes to adjust upstream process variables and subsequently assesses the effect. Sorting is an important form of automation, but it does not by itself demonstrate feedback control of food quality. The return arrow in Figure 1 represents this additional requirement for outcome information and controlled improvement.

### 3.2. Select the Modality for the Food Mechanism

RGB imaging captures visible reflectance, including color, texture, shape, and surface features. Its natural applications include appearance grading, visible-defect localization, dimensions, and package inspection. Its limitations follow from the same measurement principle: internal composition and hidden defects are not directly observed. In fermented cocoa, for example, imaging a cut surface supports comparison with cut-test grades [9]; this is a destructive sample-preparation workflow despite contactless image acquisition. “Nondestructive” should describe the complete analytical workflow, rather than the camera alone.

Multispectral imaging measures selected wavelength bands, whereas hyperspectral imaging (HSI) acquires a spectral–spatial data cube. Reflectance can contain information associated with pigments, water, tissue structure, and other matrix constituents. However, a statistical prediction of a chemical endpoint does not establish selective molecular detection. Absorption, scattering, surface geometry, and correlated biological changes can contribute simultaneously. The relevant question is whether the image–endpoint relationship survives variations that occur independently in production. The food-HSI literature provides the measurement context for these distinctions [4,5].

Postharvest respiration, ethylene-mediated ripening, pigment turnover, water loss, and cell-wall or membrane changes can alter visible reflectance and near-infrared scattering at the same time. Temperature, cultivar, maturity, and storage atmosphere may therefore create a useful but non-specific spectral association. In meat and seafood, pigment oxidation, protein and water changes, and packaging atmosphere can modify appearance independently of a target pathogen. Microbial succession depends on the initial community, temperature, oxygen availability, and competition; a spoilage-associated optical signal cannot be assumed to track every pathogen or toxin. These mechanisms motivate crossed challenge designs rather than claims of selective detection from wavelength importance alone [4,5].

Fluorescence can enhance contrast associated with endogenous fluorophores or introduced markers, while thermal imaging describes surface temperature patterns influenced by emissivity and heat exchange. Both require endpoint-specific validation: fluorescence is not automatically specific to a toxin or organism, and surface temperature is not automatically a measure of internal thermal treatment. X-ray attenuation provides information on internal structure and material contrast; it depends on composition, thickness, and energy as well as density. CT adds volumetric reconstruction but generally imposes greater acquisition and computational demands. X-ray segmentation of pear disorders and walnut inspection illustrate the value of penetrating measurements [10,11].

Depth imaging and 3D reconstruction add geometry. They can support volume estimation, portioning, and robotic presentation, but conversion from volume to mass requires a validated density relationship. Structured-light measurements of apples demonstrate the benefit of adding height information to projected area [12]. Wet or reflective surfaces, occlusion, and calibration remain relevant. Table 1 summarizes the measurement domains and the principal conditions that should bound each modality’s use.

Sensor choice is consequently an experimental question. Development should compare the simplest plausible measurement with additional bands, views, or modalities under the same independent test conditions. Extra information is valuable when it improves the intended decision sufficiently to justify acquisition time, calibration, maintenance, and cost. A multispectral production instrument derived from an HSI development study requires its own validation because illumination bandwidth, detector response, and spatial sampling change when the hardware changes.

### 3.3. Match the Prediction Unit to the Decision Unit

Classification is appropriate when an isolated item receives one label. Detection provides locations for discrete defects or foreign objects, while segmentation is preferable when extent, boundary, or trimming area matters. Regression preserves continuous information for attributes such as firmness, soluble solids, mass, or remaining time to a defined ripeness endpoint. Tracking connects observations across time so that multiple views of one product lead to one correctly timed decision. Convolutional networks, one-stage detectors, and encoder–decoder segmentation provide established computational building blocks [13,14,15,16]. Their relevance depends on the measurement task, not on architecture novelty alone.

The prediction unit can differ from the experimental unit. A kernel may generate thousands of spectra; a package may generate several frames; a fillet may generate many patches. These derived observations can improve spatial coverage, but they do not increase the number of independent physical samples. Performance should be aggregated at the level at which action occurs, with an explicit rule for combining pixels, views, or frames. For lot release, an additional sampling model is needed because item-level performance does not establish coverage of the whole lot.

Reference endpoints also require alignment. Imaging the peel of an intact fruit and analyzing a small juice subsample may introduce spatial mismatch. Assigning a freshness label by storage day can encode elapsed time rather than microbial or sensory condition. Appropriate references include calibrated dimensional measurements, defined grading protocols, validated compositional methods, sensory procedures, or microbiological testing suited to the claim. Their uncertainty, timing, and spatial correspondence should be reported. Improving label quality can provide a more defensible gain than increasing network complexity.

### 3.4. Learning When Defects Are Scarce

Transfer learning can reduce annotation requirements, while self-supervised approaches learn representations from unlabeled data. Masked reconstruction and teacher–student learning provide general strategies for this purpose [17,18]. Promptable segmentation may assist annotation, but its output still requires inspection against food-specific boundaries [19]. These methods should be compared with simpler baselines using the same independent samples and annotation budget.

Anomaly detection is attractive when normal production is abundant and defects are rare. A self-supervised HSI study reported a mean area under the receiver-operating-characteristic curve of 0.923 across strawberry and blueberry anomaly tasks [20]. This supports discrimination within the evaluated data, but a ranking score does not specify how many acceptable items will trigger an alarm. Unknown-defect testing must withhold defect categories during development and threshold selection. Otherwise, a purported open-set evaluation may still reflect prior knowledge of the anomalies.

Synthetic augmentation and multimodal fusion introduce additional assumptions. Generative augmentation has been investigated for defective coffee beans [21], and RGB–HSI fusion achieved an F1 score up to 0.90 across six papaya maturity stages [22]. Synthetic defects must be challenged with real defects; fusion must outperform each single modality on the same held-out units. Augmentation should also respect food mechanisms: broad hue changes may invalidate a color-based maturity label, and geometric transformations may remove orientation cues needed for a seal inspection. More training images are useful only when their variation remains compatible with the intended measurement.

## 4. Comparative Evidence Across Food-Inspection Endpoints

### 4.1. Produce: Appearance, Ripeness, and Internal Condition

Produce studies show why recognition accuracy and production relevance must be considered together. A tomato inspection system combined image processing with support-vector classification for defect discrimination and grading [23]. Such approaches remain appropriate when features have a clear physical interpretation and acquisition is controlled. For apples and bananas, a low-cost deep-learning system reported test accuracies of 99.2% and 98.6%, respectively, but accuracies of 96.7% and 93.8% on actual samples [24]. The additional live-sample test is valuable because it exposes a change in acquisition conditions; the results do not establish performance for all cultivars or seasons.

Multiple views address a different limitation: one image cannot show the entire surface. Citrus sorting that combined detection and tracking on rotating fruit connected visual findings to removal [25]. This improves the relevance of the experimental setup to sorting, but complete-surface coverage and item identity still require verification. A correct detection followed by rejection of the neighboring fruit is a system failure even if the image-level prediction is correct.

HSI studies on cherry tomatoes and avocados illustrate continuous quality prediction [26,27]. The avocado study reported a mean per-image root mean square error of 1.17 days to ripeness from 80 fruit followed longitudinally, producing 551 fruit images and 44,096 sub-images [26]. Its sub-image allocation placed patches from each source image into training, validation, and test sets. Thus, correlated observations crossed the evaluation boundary, and the reported error should not be interpreted as performance on independent unseen fruit. For distribution planning, a new test must hold out entire fruit and storage batches and evaluate errors near shipment cutoffs under independent storage histories.

Physical state changes optical detectability. Kiwifruit bruising experiments showed performance differences with maturity and model choice [28], while pre-symptomatic gray-mold detection achieved 96.0% evaluation accuracy on experimentally inoculated fruit [29]. These studies establish useful contrasts under controlled conditions. Commercial evaluation should include naturally occurring damage and infection, overlapping disorders, and normal product variation. Inoculation or impact treatment can otherwise be confounded with the desired biological signal.

External testing offers more direct evidence of transfer. An apple HSI study used 96 samples collected in 2023 for development and 48 samples from 2024 for temporal external validation. The two datasets included the same reported cultivar/origin groups; the study described one laboratory HSI system and did not report independent validation on another instrument or at another imaging site. Reported temporal external-validation coefficients of determination were 0.829 for vitamin C, 0.779 for soluble solids, and 0.835 for soluble protein [30]. This is evidence of temporal transfer within the sampled domain. The modest external sample size and indirect spectral relationships still limit claims about previously unseen cultivars or origins, new instruments or imaging sites, and analyte specificity. Predictive success across one year boundary should motivate broader validation rather than be described as universal industrial readiness.

### 4.2. Grains, Nuts, and Fermented Products: Grade Is Not Hazard Concentration

For dry particulate foods, inspection must reconcile small targets, variable presentation, and high material flow. RGB analysis has been applied to walnut foreign objects and rice mildew [31,32]. Cocoa cut-test classification shows a different use: quantifying a defined fermentation-related visual grade [9]. These endpoints may support commercial sorting or process assessment, but they should not be interpreted interchangeably. A cut-test grade does not directly measure flavor, and visible fungal growth does not determine toxin concentration.

This distinction is illustrated by near-infrared HSI classification of aflatoxin B1 categories in individual maize kernels. The study used prepared concentration groups and reported 95.56% overall accuracy and 88.67% accuracy in independent validation [33]. The latter result is more informative about reproducibility across acquisition occasions, but the preparation procedure remains part of the intended-use boundary. Natural contamination differs in fungal ecology, toxin distribution, kernel damage, and moisture. A chemical-safety claim requires comparison with an appropriate reference assay on naturally contaminated samples, including concentrations near the decision limit. Kernel sorting must also be evaluated against toxin concentration in the accepted lot because class accuracy alone does not establish a reduction in exposure.

Penetrating imaging can address defects concealed by shells. A lightweight X-ray walnut system reported a mean average precision at an intersection-over-union threshold of 0.5 (mAP@0.5) of 0.9869 and inference time of 11.9 ms per image, with an integrated conveyor/pneumatic sorting test and output inspections in three collections of at least 500 nuts each [10]. This provides operational evidence beyond inference timing. However, the reported ejection-precision calculation describes purity in the acceptance box, not defect-removal sensitivity. Cut-open and resealed training specimens, unresolved nut-level grouping, and the short production evaluation limit claims about transfer and sustained benefit. The study concerns structural quality, not direct demonstration of chemical or microbial safety.

Recent tea research included external scenes with changed illumination and mixed contaminants, as well as deployment at a tea production line [34]. However, the reported main dataset was augmented from 836 original images to 9196 images before random test-set allocation, creating a risk that related augmentation families crossed partitions. Its 120 subsamples also do not represent 120 independent source lots. External scenes and edge inference add operational information, but their test-unit lineage and removal outcomes must be evaluated separately; neither the main benchmark nor on-device inference establishes sustained contaminant removal benefit.

### 4.3. Meat and Seafood: Composition, Spoilage, and Foreign Material

Meat appearance reflects several interacting processes, including pigment state, water distribution, tissue structure, and surface condition. Species and adulteration studies using spectral–spatial analysis or color imaging demonstrate that discrimination is possible within defined preparation conditions [35,36]. The relevant transfer challenges include fat content, grinding, freezing and thawing, packaging, and source material. A model that separates mixtures prepared in one experiment may exploit formulation or preparation differences that do not remain stable in commercial products.

Freshness prediction requires a similarly explicit endpoint. Multispectral imaging has been used to predict microbial, sensory, and total volatile basic nitrogen (TVB-N) indicators in refrigerated trout [37]. In another trout study, an HSI-based network predicted TVB-N with a held-out prediction-set R^2^ of 0.853 and root mean square error of prediction of 3.159 mg N/100 g [38]. The study obtained 210 subsamples from 40 fish and allocated 140/70 subsamples to calibration/prediction; separation by parent fish was not established in the reported split. This was a laboratory prediction set, not a new-site external validation. Its comparison with published chemometric studies did not establish a consistent advantage for deep learning. Absolute error, reference-method agreement, and stability across storage conditions are consequently more meaningful selection criteria than model family.

TVB-N and sensory deterioration are quality indicators; they do not establish the absence of a particular pathogen. An inspection system intended to support safety decisions must demonstrate the specific hazard relationship needed for that use. Parasite detection in cod provides an example of a directly localized target. A study under industrial conditions reported a nematode detection rate of 73%, compared with a conventional manual-inspection rate of approximately 50% [39]. The latter is a comparator described in the paper, not evidence here of a randomized paired comparison. The result demonstrates useful improvement potential while also showing that substantial residual misses remain.

Foreign-material studies on poultry reveal how target size changes performance. Fusion of visible–near-infrared and short-wave-infrared HSI detected all evaluated opaque 5 × 5 mm pieces, whereas detection of 2 × 2 mm polymer, wood, and metal pieces was 95%, 95%, and 81%, respectively [40]. Transparent and semitransparent materials were excluded from model evaluation because their spectra were not sufficiently distinguishable. The reported 100% therefore applies only to the evaluated larger opaque pieces, not all foreign materials. Spatial resolution, partial coverage by tissue, orientation, and spectral similarity should be challenged explicitly. Detection of material placed on the surface does not establish detection of the same material embedded inside a fillet.

Semisupervised learning subsequently achieved 96.5% object-level foreign-material detection using near-infrared HSI [41]. Hardware optimization of this approach reduced inference time by up to fivefold and model size by 50%; compatibility with 140–250 birds per minute was evaluated through simulations using hypothetical line-scan cameras [42]. Together these studies form a useful sequence from analytical contrast to computational feasibility. The remaining demonstration is synchronized acquisition, detection, and correct removal under representative production conditions, with false alarms and missed objects measured at the product level. The compression benchmark reused 48 HSI images from 12 fillets, including images used during model development, so its timing gains should not be interpreted as an independent estimate of detection generalization.

### 4.4. Packaging and Process Geometry: Strong Integration, Precise Denominators

Packaging offers repeatable geometry, a defined inspection station, and a clear accept–reject action. A pizza-package study evaluated closure and seal inspection across three brands with two toppings per brand, reporting mean classification precision of 99.87% and twice the manual throughput [43]. These are relevant operational outcomes within the studied setting. However, an optical seal defect remains a surrogate for a loss of package integrity unless linked to leakage or another appropriate functional reference. Changes in film, print, product placement, or seal design require evaluation of that relationship.

Thermoformed-package inspection provides a particularly instructive denominator example [44]. In the study’s production test with process problems, the published counts were 206 true positives, 3 false negatives, 13 false positives, and 4082 true negatives. The quoted 0.07% missed-product fraction is 3/4304. Recalculation from the same counts gives defect sensitivity of 206/(206 + 3) = 98.56%, rather than 99.93%. Both quantities are useful, but they answer different questions. The study demonstrates production integration; its interpretation requires distinguishing defect sensitivity from the proportion of all products that escape detection. Table 2 preserves that distinction.

**Table 2:** Selected empirical examples and the specific claims supported by their evaluation. Table S1 gives the associated sample counts, references, grouping, environments, evidence dimensions, and qualitative limitations.

Endpoint/Reference	Measurement and Evaluation	Reported Result	Evidence Boundary/Interpretation
Apple and banana grading [24]	RGB; image tests and live samples	Image tests: 99.2%/98.6%; live: 96.7%/93.8% accuracy	Live acquisition exposes a performance change; broader production transfer remains a separate claim.
Avocado ripeness [26]	HSI; 80 physical fruit; 551 longitudinal images; 44,096 sub-images	Mean per-image RMSE: 1.17 days to ripeness	Sub-images from each image entered all splits; reported error does not establish unseen-fruit generalization.
Apple composition [30]	2023 development, n = 96; 2024 temporal external validation, n = 48	Temporal external-validation R^2^: 0.829 vitamin C; 0.779 soluble solids; 0.835 soluble protein	Shared cultivar/origin groups across years; independent instrument or imaging-site transfer not reported; no universal chemical selectivity implied.
Maize aflatoxin categories [33]	NIR-HSI; kernels dosed with standard solutions	95.56% overall accuracy; 88.67% independent-validation accuracy	Prepared concentration groups; natural contamination and accepted-lot toxin burden require separate tests.
Walnut internal defects [10]	X-ray detector; conveyor/pneumatic sorting; three output collections of at least 500 nuts each	mAP@0.5 = 0.9869; 11.9 ms/image inference	Output checks add operational evidence; reported ejection precision is accepted-box purity, not removal sensitivity.
Tea foreign objects [34]	RGB; 836 images augmented to 9196 before random test allocation; edge deployment	Raspberry Pi 5: 11.3 frames/s; deployment mAP = 98.92%	Risk of augmentation-family leakage; external scenes and edge timing do not establish correct removal.
Trout TVB-N [38]	HSI; 40 fish yielding 210 subsamples; 140/70 calibration/prediction	R^2^ = 0.853; RMSEP = 3.159 mg N/100 g	Parent-fish grouping unresolved; laboratory quality prediction, not pathogen absence or new-site validation.
Cod nematodes [39]	Spectral–spatial deep learning under industrial conditions	73% detection; approximately 50% manual comparator cited	Residual misses remain; the comparator is not treated here as a randomized paired comparison.
Poultry foreign objects [40]	12 fillets; 96 HSI images; placed surface objects; opaque materials evaluated	5 × 5 mm: 100%; 2 × 2 mm: polymer 95%, wood 95%, metal 81%	Transparent/semitransparent targets excluded; material/size-specific detection, not embedded-object detection or removal.
Poultry inference optimization [42]	48 reused HSI images from 12 fillets; compression; hypothetical camera simulations	Up to 5-fold acceleration; 50% smaller model; simulated 140–250 birds/min	Computational benchmark includes development images; not independent accuracy or prospective removal validation.
Thermoformed seals [44]	Production test with process problems; 4304 products	TP = 206; FN = 3; FP = 13; TN = 4082	Calculated sensitivity: 98.56%; missed fraction of all products: 0.0697%. These denominators differ.
Pizza-package closure [43]	Laboratory and industrial tests; three brands, two toppings each	Mean precision: 99.87%; 2× manual throughput	Operational benefit within the tested setting; optical defects require a functional integrity reference.
Bacteria versus food debris [45]	Microcolony imaging; three food matrices; *B. subtilis* validation	123/133 colonies detected; 7 false detections; precision 94.6%, recall 92.5%	Object-level performance within the assay; independent food-sample sensitivity must be established separately.

HSI, hyperspectral imaging; NIR, near-infrared; SWIR, short-wave infrared; mAP, mean average precision; mAP@0.5 uses IoU 0.5; TVB-N, total volatile basic nitrogen; RMSEP, root mean square error of prediction; TP/TN, true positives/negatives; FP/FN, false positives/negatives.

Geometry measurements can support upstream process adjustment. Color segmentation of traditional Carasau bread, for example, was developed to assess product dimensions during production [46]. Such outputs become process-control measurements only after repeatability, physical calibration, and response to process changes are established. A visible deviation may result from forming, proofing, baking, handling, or camera conditions. Root-cause identification therefore requires process context rather than an automatic assumption that the nearest adjustable setting is responsible.

### 4.5. Microbiological Imaging: Faster Readout Within a Defined Assay

Microcolony imaging changes the analytical workflow by recognizing growth before conventional endpoint reading. AI-assisted optical detection has demonstrated early bacterial detection relevant to food testing [47]. Its contribution is to culture observation and interpretation; it should not be described as direct recognition of every pathogen in an intact food image. Sampling, recovery, incubation conditions, and confirmation remain part of the method.

Food debris is a concrete source of matrix interference. A model trained on bacteria alone misclassified debris as bacteria at a reported rate of 24.2%; including chicken, spinach, and cheese debris in training improved discrimination [45]. In the food-matrix validation with fluorescently identifiable *Bacillus subtilis*, the system detected 123 of 133 microcolonies and produced 7 false detections, corresponding to mean precision of 94.6% and recall of 92.5%. These results support the value of realistic negative examples. The microcolony images were acquired after 3 h of incubation following sample preparation and plating. This is not a validated end-to-end sample-to-result time or a universal time to demonstrate food safety.

Validation should distinguish object detection from sample-level analytical sensitivity. Many colonies on one plate do not represent many independent food samples. Low initial contamination, injured cells, strain variation, mixed cultures, debris load, and recovery efficiency can change the probability that a target becomes visible. Deployment claims should report the complete time from sample preparation to decision, the reference method, the number of independent samples, and performance near the required detection level. A faster image-reading step is valuable only when the full assay remains fit for its intended use.

For an alternative microbiological method intended to support release decisions, the entire workflow—not only the image classifier—should be compared with the relevant reference method within the applicable ISO 16140 validation route [48]. The study design should cover the claimed food categories, target strains and non-target organisms, low-level fractional positives, recovery of stressed or injured cells, enrichment and incubation conditions, background flora, and matrix interference. Relative detection capability, discordant-result confirmation, and reproducibility should be reported. Laboratory implementation verification is a distinct step under ISO 16140-3 [49]; neither standard converts colony-level recall into a probability of detecting contamination in an entire lot.

## 5. Organizing Validation Evidence for the Intended Use

### 5.1. Preserve Independence Throughout Model Development

Data leakage can arise before model fitting, during preprocessing, or through repeated access to a test set. Its consequences for scientific reproducibility extend beyond food imaging [50]. In this field, the practical safeguard is a record linking every image, spectrum, patch, and augmented observation to its parent sample and acquisition group. Partitioning should occur before augmentation or patch generation. All observations from the same physical item should remain together, with higher-level grouping when the intended claim concerns new batches, seasons, or sites.

The model-development pipeline must be included in that separation. Learned normalization, wavelength selection, feature selection, class balancing, and hyperparameter tuning should use development data only. When data are limited, nested grouped cross-validation separates model selection from performance estimation. Physically defined corrections, such as a white-reference calibration acquired for each imaging session, are different from learning a transformation from the combined training and test samples; both procedures should be documented. A test set ceases to be an independent final test when its results repeatedly guide design choices.

Temporal grouping is especially important for storage experiments. Observations from one product at different storage times share biological history. Splitting them between development and evaluation can make prediction appear more transferable than it is. Independent items alone may also be insufficient if all items from one treatment were imaged in a single session and all controls in another. Randomizing acquisition order, balancing conditions across sessions, and holding out complete batches help separate the food effect from the imaging effect.

An external test should specify what is external. A new year at the same site evaluates temporal transfer; a new camera evaluates instrument transfer; a new supplier evaluates a different product distribution. Success on one dimension does not establish success on the others. When adaptation is needed, performance before and after adaptation should be reported separately, and the adapted model requires an untouched evaluation set. These distinctions make external validation a defined experiment rather than a descriptive label.

### 5.2. Report Metrics with Their Denominators and Uncertainty

The task-dependent reporting set in Table 3 begins with independent sample counts and the confusion matrix. Classification requires class-specific sensitivity, specificity, and precision at the operating threshold. Detection requires the localization criterion, object-size distribution, and per-class recall. Segmentation requires agreement in extent or boundary when those quantities affect trimming or rejection. Regression requires absolute error in physical units, bias, residual structure, and uncertainty around the decision boundary. A global R^2^ may be high because the reference range is wide while prediction error remains unacceptable for distinguishing adjacent grades. Precision–recall curves and their area (PR-AUC), together with prevalence and the fixed operating point, are particularly informative for rare-defect and anomaly tasks. Probability models should include calibration plots and suitable numerical calibration summaries. Analytical concentration or presence/absence claims additionally require method-appropriate detection capability, quantification limits where meaningful, measurement uncertainty, repeatability, and reproducibility. A visual grade classifier does not have an analyte LOD/LOQ simply because it uses a numerical score.

Denominators must be explicit. False-negative rate among defective items is FN/(TP + FN); the fraction of all inspected items that are missed is FN/N. Similarly, false-positive rate among acceptable items differs from the fraction of rejected items that are acceptable. The packaging recalculation in Section 4.4 demonstrates why these quantities cannot be substituted. A report should also state whether the counted unit is a pixel, object, package, fillet, or lot. Averaging a metric across images can conceal poor performance on a small but important defect category.

Uncertainty should reflect the sampling design. For zero misses among n independent representative positive items with a common miss probability, the exact one-sided 95% binomial upper bound is 1 − $0.05^{1/n}$, approximately 3/n. Zero misses in 100 independent positives gives an upper bound of about 2.95%; 2995 independent positives with no misses are needed to bring this bound below 0.1%. These calculations concern conditional item-level sensitivity only. They do not account for batch clustering, spatially heterogeneous contamination, selection bias, or the probability that the lot-sampling plan captures a hazard, and are not food-safety sample-size prescriptions.

When batches or lots drive dependence, the number and allocation of independent clusters must enter the design and analysis. Suitable prespecified hierarchical models or conservative designs based on independent clusters may be needed; resampling an all-zero-error dataset alone cannot establish a valid nonzero upper error bound. Lot acceptance additionally requires a sampling plan that specifies the lot, sampling unit, increments and composites, test method, and acceptance rule. Its operating-characteristic curve should show the probability of acceptance over relevant contamination levels and distributions [51]. AQL-based producer-risk protection is not a universal food-safety criterion: the applicable plan may prioritize consumer protection. Single-kernel/image accuracy cannot establish lot conformity or reduced consumer exposure.

Rare but severe defects should be reported separately even when estimates are imprecise. Enriched challenge sets are useful for estimating sensitivity to particular materials, sizes, or defect severities, but they do not reproduce production prevalence. A complementary representative production set is needed for estimating false alarms, positive predictive value, and review workload. Reporting both sets avoids forcing one dataset to answer incompatible questions.

### 5.3. Connect Thresholds to Prevalence and Action

Production prevalence changes the practical meaning of an apparently strong classifier. Consider an illustrative population of 100,000 items with 0.1% defects, inspected at 99% sensitivity and 99% specificity. Expected counts are 99 detected defects, 1 missed defect, and 999 acceptable items incorrectly flagged. Only about 9% of flagged items would be defective. If every flag leads directly to disposal, the resulting waste could dominate the benefit. The example does not imply that sensitivity should be relaxed; it shows why specificity, confirmatory review, and staged decisions matter when defects are rare.

For quality grading only, a binary accept/reject approximation can illustrate how prevalence and error consequences affect a fixed threshold t:

(1) $$L(t)\;=\;C_{FN}\pi [1\;-\;Se(t)]\;+\;C_{FP}(1\;-\;\pi )[1\;-\;Sp(t)]$$

Here, π is the prevalence of the defined quality defect, Se(*t*) is sensitivity, Sp(*t*) is specificity, and *C*_FN_ and *C*_FP_ are quality-related error costs. Equation (1) is a teaching example with fixed costs, not a proposed safety objective or a complete production policy. A multi-action workflow instead evaluates accept, hold, confirm, rework, reject, and stop decisions, including their conditional outcomes, delay, and review capacity. Hazard-control requirements and applicable legal limits first define the admissible actions; quality cost, yield, or waste can then be optimized only within that admissible set. An unsafe release is not made acceptable by assigning it a finite cost, and no economic gain compensates for failure to meet a validated safety requirement [52].

Probability calibration can help interpret scores but does not remove this need for decision-specific validation. Temperature scaling is a useful post-processing approach for some classifiers [53], while empirical evaluations show that calibration can deteriorate under distribution shift [54]. Raw object-detection confidence and anomaly scores should not automatically be described as probabilities of food-safety failure. The threshold and any calibration procedure should be fixed before final evaluation.

An abstention route is appropriate when image quality fails, the product lies outside the validated domain, or the score is insufficient for a reliable decision. Its workload and residual risk must be measured: a model can appear highly accurate by sending many difficult cases to manual review. Reporting performance together with the fraction of items receiving automated decisions makes this trade-off visible. Low confidence should trigger a defined response, rather than silently defaulting to acceptance. The reason for abstention should be coded separately as acquisition/observability failure (for example severe occlusion or saturated glare), domain mismatch, or predictive ambiguity. Occluded or unusable inputs are uninspectable, not negative findings. Re-imaging or an alternative validated inspection route may resolve them; otherwise the product is held under the established control procedure. Report each cause, its frequency among all presented items, final disposition, and residual misses after review. Evaluation should retain non-imaged, lost, and unresolved items in the system-level accounting.

### 5.4. Use Explanations to Investigate Failure Mechanisms

Visual explanations can reveal reliance on a tray, label, background, or illumination artifact rather than the food defect. Gradient-based localization provides one established approach [55]. For spectral models, wavelength relevance can be compared with plausible optical mechanisms. However, a highlighted region or an apparently meaningful band does not establish causality, selectivity, or external validity. Correlated variables may receive convincing attributions even when their relationship changes outside the experiment.

The useful test is whether an explanation leads to a falsifiable challenge. Background replacement can probe context dependence; varying temperature independently of storage time can probe confounding; testing different packaging atmospheres can challenge a color–freshness relationship. Masking an attributed region may inform interpretation, although the masking operation itself can create unrealistic images. Explanations are therefore best used to design controlled follow-up experiments and improve data collection. They complement quantitative validation and do not replace it.

### 5.5. Interpret the Proposed Evidence Map by Intended Use

**Figure 2:**
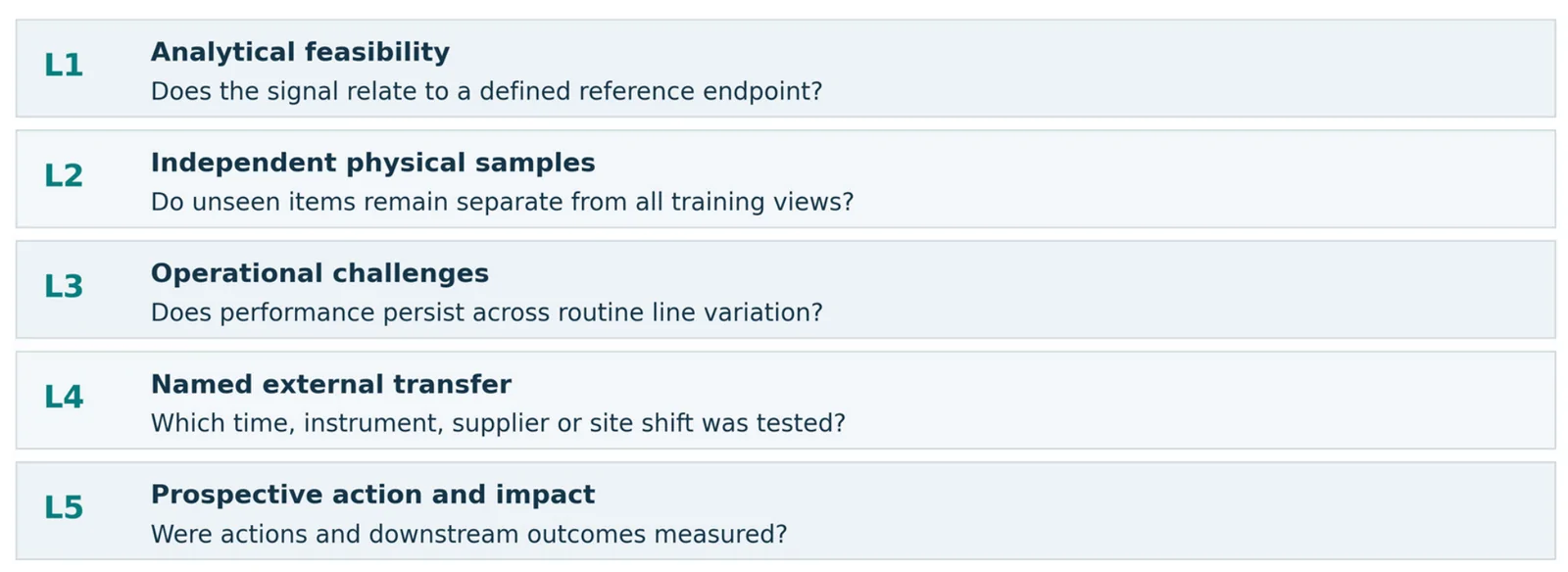
Proposed evidence map. L1–L5 are evidence-dimension labels, not cumulative levels, maturity gradations, or readiness scores. The dimensions are partly independent and should be reported separately. External transfer must name the tested shift, and prospective sorting does not establish feedback control. Safety-specific requirements apply across all dimensions; no dimension or combination of dimensions authorizes product release or demonstrates hazard absence.

Figure 2 organizes five evidence questions using the labels L1–L5. L1 concerns analytical feasibility in a restricted setting; L2 concerns unseen physical samples with derived observations grouped; L3 concerns routine operational challenges; L4 concerns a locked system under explicitly named external shifts; L5 concerns prospective actions and downstream consequences. L1–L5 are evidence-dimension labels, not cumulative levels, maturity gradations, or readiness scores. A study can address several dimensions incompletely, and strong evidence on one dimension cannot replace missing evidence on another.

The organization is an author-derived synthesis of the endpoint mismatches in Section 4, sample-independence concerns [50], uncertainty under shift [54], and food-control principles [52]. No Delphi exercise, expert consensus, inter-rater reliability study, construct validation, or predictive validation of the map was performed. It is offered as a discussion and study-design aid, not a recommended regulatory framework. Table 4 maps selected concepts to TRIPOD + AI, CLAIM 2024, and STARD-AI [56,57,58]. Those documents concern healthcare prediction or diagnostic reporting, not food-device approval; the mapping identifies transferable reporting principles and does not assert equivalence or compliance. If the map is developed into a decision instrument, operational definitions, independent assessor testing, and prospective association with relevant production outcomes would be required.

### 5.6. Apply a Separate Safety Gate

A shared vocabulary for evidence does not imply a shared acceptable risk. Appearance grading can be assessed against a defined commercial grade and its error tolerances. By contrast, safety-supporting decisions require an independently specified hazard, validated reference endpoint, applicable critical or legal limit, representative matrix and challenge conditions, and a justified upper bound on the relevant error or residual hazard. Their acceptance criteria must be determined through the existing hazard-control system and applicable requirements, not inferred from the evidence-dimension label or from another commodity [52].

For mycotoxin sorting, verify chemical concentrations in representative accepted lots and the sampling-plan performance, including material near the applicable limit; kernel classification is only a component. For foreign bodies, validate detection plus removal by material, size, location, and product presentation, and verify the accepted stream. For package integrity, pair visible or thermal seal findings with an appropriate functional leak/barrier reference and the claimed storage conditions. For pathogen detection, validate the complete analytical method, including recovery and confirmation, as described in Section 4.5. These endpoint-specific gates supplement rather than replace the evidence dimensions. Food-safety failure triggers holding, corrective action, or the established fallback even when quality and throughput targets are met.

### 5.7. Prespecify External Transfer Acceptance

Time, instrument, supplier, and site transfer are separate claims. Specify which factors change, which remain fixed, the locked model/threshold/calibration, and the intended domain before external testing. Use separately reported strata and, where practical, crossed or paired designs so that simultaneous changes are not mistaken for a single transfer effect. Each claimed domain requires a justified sample size based on the endpoint, expected error, required precision or power, defect prevalence, and number of independent batches or sites; repeated images do not substitute for these units.

Acceptance must be tied to intended use. For sensitivity, prespecify a minimum acceptable value and require the appropriate one-sided confidence bound to exceed it; a non-inferiority comparison can additionally bound degradation relative to a valid comparator using a prespecified margin. For quantitative measurements, specify allowable bias and error in physical units around the decision limit. Equivalence requires the relevant confidence interval to lie wholly within a prespecified equivalence interval; a non-significant difference alone is insufficient. Margins should come from grade tolerances, measurement requirements, or hazard analysis—not the observed test results. There is no defensible universal percentage degradation for all food endpoints. Adaptation data and post-adaptation evaluation data must remain separate, and safety criteria retain priority over relative model comparisons.

## 6. Integrating Inspection with Food-Process Control

### 6.1. Validate Acquisition, Identity, and Actuation Together

The deployed system includes optics, product presentation, preprocessing, model, threshold, communication, and actuator. Illumination geometry, exposure, camera distance, lens cleanliness, and motion blur affect what the model receives. HSI additionally requires appropriate radiometric checks and attention to wavelength response and temperature. Reference targets and defined acceptance criteria can detect some acquisition changes before they affect product decisions. Camera settings and preprocessing versions should therefore accompany the model version in validation records.

Product presentation determines coverage. Singulation, rotation, belt loading, and overlapping products change whether a target is visible and whether the decision can be assigned correctly. Challenge tests should follow known objects through acquisition, tracking, and removal, with verification downstream of the ejector. An item missed because it was not imaged remains an inspection-system miss, even if it never enters the model’s test denominator. Similarly, incomplete removal must not be counted as successful control merely because a detection command was issued.

Total response time includes exposure or scanning, transfer, preprocessing, inference, decision communication, and physical actuation. Mean inference speed cannot establish timing reliability; latency variation, synchronization, and worst-case delays are relevant when closely spaced products move toward an ejector. The tested hardware, image size, batching assumptions, and simultaneous tasks should be stated. Throughput should ultimately be reported as inspected product flow with verified actions, rather than only frames per second.

The tea study illustrates hardware dependence particularly clearly: approximately 340.6 frames per second in the computational benchmark corresponded to 11.3 frames per second on a Raspberry Pi 5 at the production station, with a reported deployment mAP of 98.92% [34]. The device result is directly useful for engineering capacity. Its interpretation still depends on how frames map to products and how many views each product requires. Comparisons that omit the hardware context can exaggerate deployment capability by an order of magnitude.

Table 5 translates the conceptual architecture into a proposed implementation-validation checklist. Each trial should prespecify its operating envelope, denominators, acceptance limits, sampling plan, responsible decision-maker, and failure-response classification according to intended use and hazard analysis. The checklist is a design aid; its completion is not evidence that a particular installation has passed.

### 6.2. Distinguish Monitoring, Verification, and Revalidation

Food processes change with ingredients, suppliers, equipment wear, sanitation, formulations, and storage conditions. A decline in model confidence may indicate product change, sensor change, or both. Conversely, a confident model can fail under a new distribution. Monitoring should therefore combine input-quality checks and score distributions with periodic reference measurements and downstream observations. Input drift is an investigation signal; it does not by itself prove that decision performance has deteriorated.

Three activities serve different purposes. Routine monitoring establishes whether the inspection station is operating within its approved conditions. Verification checks that the implemented control continues to function as intended. Revalidation evaluates whether evidence remains adequate after a material change in product, process, sensor, model, or threshold. The distinction is consistent with the broader food-hygiene and hazard-control principles published by the Food and Agriculture Organization of the United Nations (FAO) and the World Health Organization (WHO) [52]. The specific checks proposed here adapt that logic to image acquisition and machine-learning components; they are not additional requirements stated by Codex for AI systems.

Reference testing should include accepted products as well as rejected or disputed ones. Reviewing only alarms leaves high-confidence false negatives largely invisible. Stratified sampling across suppliers, shifts, and product conditions can provide more informative evidence than a convenient sample of obvious rejects. For regression models, reference residuals should be followed for bias as well as average error. Changes in the reference method itself require investigation before attributing drift to the imaging model.

Table 6 links common failure modes with practical controls and escalation triggers. Retraining should follow diagnosis, not act as the default response to every alarm. A dirty optical window requires maintenance; inconsistent labels require reference-method correction; previously unrepresented product variation may justify additional training data. Updated models should be evaluated offline against appropriate retained and new challenge sets, with documented acceptance before deployment. Reusing a benchmark repeatedly for tuning requires a refreshed independent evaluation to preserve its evidential value.

### 6.3. Establish Process Benefit with a Prospective Comparison

An initial prospective phase can record predictions while established controls remain in place. This phase estimates production prevalence, disagreement, false alarms, and timing behavior without attributing benefit to actions the system has not yet taken. Threshold development should be separated from the subsequent evaluation phase. Once the rule is fixed, an intervention study can compare the defined inspection workflow with a suitable baseline.

Outcome selection should reflect the food problem. Relevant measures include defect escape in accepted product, false rejection of acceptable product, confirmed contaminant burden, yield, rework, inspection labor, downtime, and waste. Safety performance should be evaluated separately from quality and economic outcomes. Increased rejection is not necessarily improved safety, and reduced waste is not necessarily desirable if the accepted stream contains more hazardous defects. Denominators, observation duration, product mix, and concurrent process changes must be reported.

Where feasible, paired lines, randomized time blocks, or staged introduction can strengthen causal interpretation. Comparisons before and after installation are more vulnerable to changes in suppliers, season, staffing, and throughput. Artificial challenge materials can test detection and removal, but should be combined with normal production because their prevalence and placement differ from naturally occurring problems. Claiming a reduction in recalls or consumer risk requires evidence beyond a short pilot with no observed incidents.

### 6.4. Close the Loop Within a Validated Process Envelope

The following baking loop is a proposed architectural design example, not an experimentally validated closed-loop system or a field trial. Surface-color distribution might inform a bounded change in heating conditions, but the review does not report testing of that feedback intervention. The Carasau bread study [46] concerns image segmentation and dimensional inspection; it is not evidence that the proposed color-based feedback loop improves production. Such a loop would require separate validation against moisture, texture, sensory acceptance, transport delay, and any independently controlled safety endpoint. More uniform color alone would not demonstrate improved overall product quality.

Feedback design must account for transport delay, residence-time distribution, actuator limits, and the possibility that the measurement changes after the process intervention. Adjusting a process can alter the very data distribution on which the model was trained. Excessively rapid responses to noisy visual measurements may produce oscillation, while optimizing one visible attribute may worsen another attribute that is not measured. Stable operation requires appropriate filtering, update intervals, and independent constraints.

**Figure 3:**
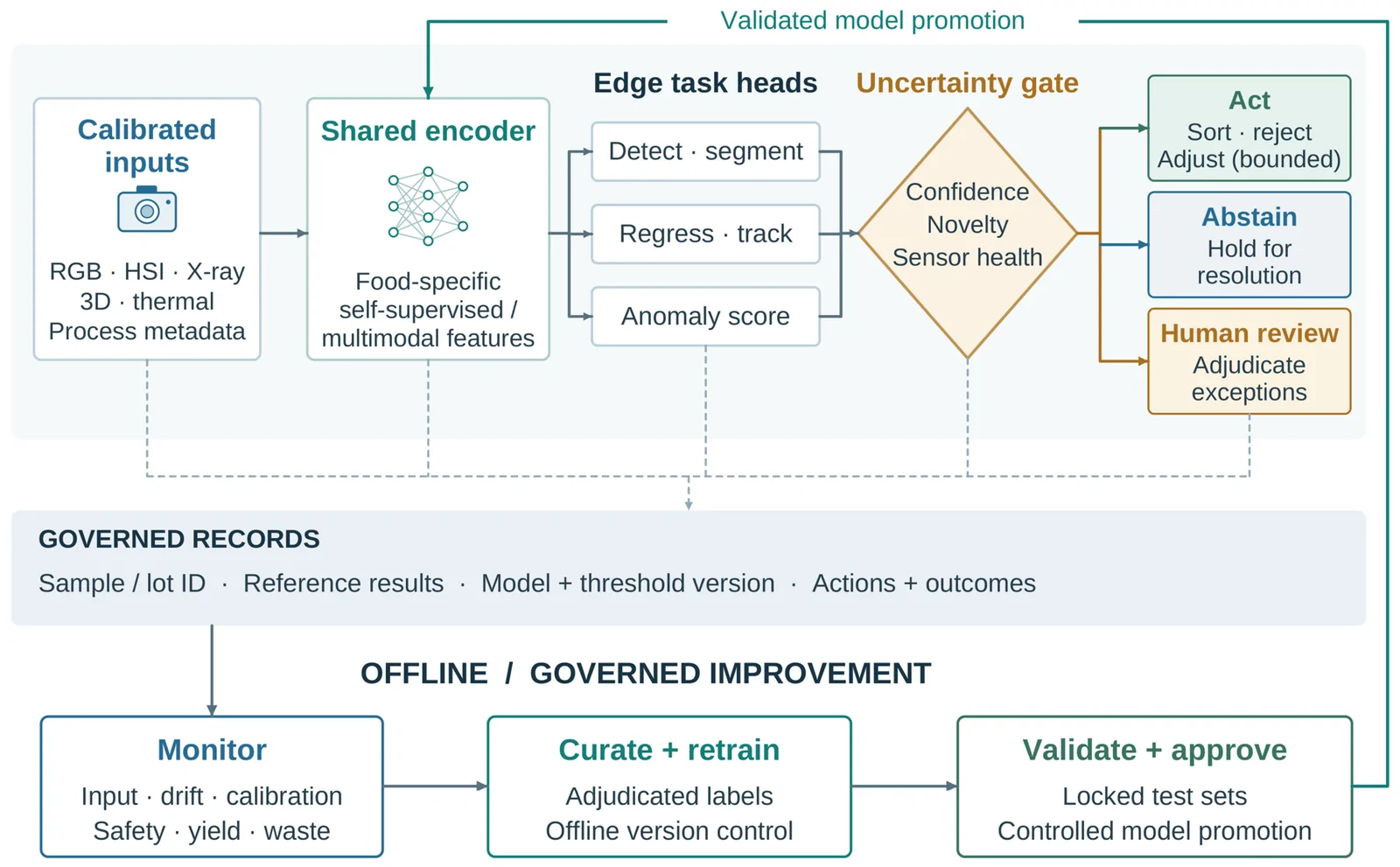
Proposed architecture separating operational inference from controlled improvement. Calibrated inputs feed task-specific predictions; sensor health and uncertainty guide action, holding, or human review. Traceable records connect products, reference measurements, model versions, actions, and outcomes. Updated models require evaluation and approval before deployment. A shared encoder is an optional implementation. RGB, red–green–blue; HSI, hyperspectral imaging; 3D, three-dimensional; ID, identifier. Solid arrows denote operational flow; dashed links denote records supporting traceability. This is a conceptual proposal, not an implementation validation result; Table 5 lists corresponding empirical checks.

Figure 3 proposes an architecture that separates rapid operational inference from slower model improvement. Sensor checks and uncertainty handling determine whether to act, hold, or request review; traceable records connect predictions to products, decisions, reference results, and outcomes. A shared representation is one possible implementation, not a prerequisite. Changes to models and decision rules occur through a controlled evaluation process. For hazard-control applications, validated critical limits and corrective-action procedures retain priority over optimization objectives [52].

## 7. Research Priorities for Transferable Food Vision

The first priority is better test design. New datasets should retain physical-sample and acquisition-group identifiers and support challenges across batches, time, and instruments. The HSI-AgriFoodAnomaly dataset provides 147 calibrated cubes of an oat–chocolate mixture with foreign-object annotations and cube-level partitions [59]. This is a useful reproducibility resource. Cube separation should nevertheless be assessed against physical scene and object reuse, and one mixture under one acquisition design cannot represent all food matrices. Shared benchmarks should make these boundaries visible and include representative negatives, difficult defects, and reference measurements where available.

The second priority is mechanistic robustness. Experiments should vary likely confounders independently of the endpoint, including temperature, moisture, product geometry, lighting, and packaging. Spectral models should be challenged where a correlated appearance change no longer tracks chemical concentration. Imaging of natural defects is needed alongside controlled defect preparation. The aim is to identify conditions under which the measurement relationship is stable, rather than to infer mechanism from a high predictive score or a feature-attribution plot. For example, a ripeness experiment should cross cultivar and harvest maturity with temperature and atmosphere, randomize acquisition order, and hold out whole harvest/storage batches. A toxin study should challenge naturally contaminated and prepared kernels across moisture, fungal damage, and toxin concentrations that are not perfectly correlated, with independent chemical references. Mechanistic challenges should include matched samples with similar appearance but different target levels, and different appearance at similar target levels. Performance should be reported by challenge condition rather than as a pooled score alone.

The third priority is economical use of labels and sensors. Self-supervision, transfer learning, annotation assistance, and multimodal representations can reduce development effort, but their benefit should be measured through the number of independently validated samples, annotation time, and decision performance. Sensor reduction should compare complete devices rather than selected bands from the same hyperspectral cube alone. Models should also be tested with a missing or degraded modality so that an auxiliary sensor failure has a defined consequence. Foundation models are a research opportunity; their size or general-image performance does not establish calibration for food inspection.

The fourth priority is prospective evidence of intervention benefit. A stronger contribution may come from showing sustained reduction in confirmed defect escapes at an acceptable false-reject burden than from a small gain on a reused benchmark. Such studies should document model and threshold versions, normal production variation, maintenance, operator overrides, and accepted-product checks. For closed-loop claims, the effects of feedback and process adjustment must be demonstrated in addition to detection performance. This shift would connect advances in computer vision to the outcomes that matter in food science: reliable measurements, controlled hazards, consistent quality, and efficient use of edible material.

## 8. Conclusions

AI-enabled computer vision becomes a useful food-inspection technology when the observed signal is linked to a defined endpoint, independent validation matches the intended operating conditions, and the resulting action is verified. The strongest contribution of imaging is often frequent, spatially resolved measurement, but neither high accuracy nor rapid inference establishes food safety or process benefit. Quality grading, chemical prediction, hazard detection, and package-integrity assessment require different references and decision criteria. Progress should be judged through explicit metric denominators, uncertainty, transfer to new production, and verified effects on accepted-product quality, defect escape, and waste. Automated sorting and closed-loop process control should remain distinct claims. A complete validation strategy connects food mechanism, sensor performance, model behavior, decision thresholds, actuation, and lifecycle checks; this connection is the essential step from promising image analysis to dependable food-process control. The five-dimension evidence map is a provisional synthesis, not a validated readiness scale, consensus standard, or authorization for safety decisions. The selected literature and qualitative appraisal identify recurring failure modes but cannot establish their frequency across the industry. Prospective endpoint-specific validation remains necessary before an installation or feedback intervention can claim operational benefit.

## Figures and Tables

**Table 1:** Imaging modalities, measurement domains, and limits relevant to food inspection.

Modality	Measured Contrast	Suitable Inspection Task	Main Validation Boundary
RGB	Visible reflectance; color, texture, and shape	Surface grading; visible defects; labels and seals	Illumination and coverage; chemical predictions are indirect
Multispectral	Reflectance or emission in selected bands	Targeted composition, condition, or foreign-material screening	Band choice and calibration are endpoint-specific
Hyperspectral	Spectral–spatial variation across contiguous bands	Composition modeling; subtle defects; material discrimination	Matrix correlations, scattering, temperature, and instrument transfer
Fluorescence	Emission following controlled excitation	Fluorophore-related changes; residues or marker contrast	Excitation, background, and matrix specificity
Thermal	Surface radiance related to temperature and emissivity	Heating or cooling patterns; process nonuniformity	Surface–core relationship; emissivity, airflow, and timing
X-ray/CT	Energy-dependent attenuation; internal structure	Internal defects, voids, and contrasting foreign objects	Thickness, composition, spatial resolution, and acquisition time
3D/depth	Surface geometry and spatial dimensions	Volume, portion geometry, and robotic positioning	Occlusion and calibration; mass also depends on density

RGB, red–green–blue; CT, computed tomography; 3D, three-dimensional. Modalities are compared by the information they measure, not by an overall performance ranking.

**Table 3:** Proposed task-dependent reporting elements linking model evaluation to food decisions. Items apply when meaningful for the claimed endpoint and do not constitute a universal approval checklist.

Task	Statistical Evidence	Design Information	Decision Metric
Classification	Confusion matrix; class sensitivity, specificity, precision; PR-AUC; calibration plots if probabilistic; intervals	Independent positives/negatives and clusters; prevalence; grouped split; locked threshold; external domains	Escapes, false rejects and review workload at the operating point
Detection	Recall by class/size/position; PR curves; mAP with IoU; uncertainty	Annotation and matching rules; independent objects/items; occlusion; image-to-item mapping	Correct-object removal; wrong-item rejection; accepted-stream residuals
Segmentation	Overlap; boundary/area error; repeatability and uncertainty	Reference masks and ambiguity; grouped samples; cross-operator/day testing	Trimming margin, yield and residual defect area
Regression	MAE/RMSE in units; bias; residuals; prediction-interval coverage; repeatability/reproducibility	Reference uncertainty; range; independent domains; error near limits; applicable LOD/LOQ	Misrouting near specifications; uncertainty-aware hold zone
Anomaly detection	PR-AUC and ROC-AUC with prevalence; fixed-threshold sensitivity/false alarms; threshold stability	Withheld defect categories; grouped external tests; threshold chosen on development data only	Novel-defect capture; review fraction; performance versus automated coverage
Analytical assay claim	Matrix-specific detection capability; LOQ where relevant; measurement uncertainty; repeatability/reproducibility	Applicable reference-method comparison; recovery; interference; low-level challenges and independent samples	Sample-level false-negative/positive risk; link to lot-sampling plan
Abstention route	Rates by cause among all presented items; reinspection outcomes; residual error after review	Separate uninspectable input (occlusion/glare), domain mismatch and predictive ambiguity; include lost/unresolved items	Defined re-image, alternate test, hold or stop; no default acceptance
Tracking and control	Identity error; latency distribution; removal success; stability	Synchronization; downstream confirmation; independent batches; prospective intervention design	Accepted-product defects/hazards; false rejects; yield; downtime; waste

MAE, mean absolute error; RMSE, root mean square error; mAP, mean average precision; IoU, intersection over union; PR-AUC, area under the precision–recall curve; LOD/LOQ, limit of detection/quantification. Report physical-sample and batch counts, dependence-aware uncertainty, threshold version, and operating domain.

**Table 4:** Conceptual mapping to selected reporting guidance. The mapping is not a claim of checklist compliance or food-safety approval.

Evidence Question	Relevant Reporting Guidance	Food-Specific Addition and Boundary
L1 Signal and endpoint	CLAIM 2024: acquisition, reference labels, and model description [57]	Report matrix, optical mechanism, reference uncertainty, and sampling correspondence. Reporting completeness does not validate specificity.
L2 Independent samples	TRIPOD + AI and CLAIM: data sources, partitions, performance, and uncertainty [56,57]	Group frames, patches, and spectra by physical item and batch. Report independent counts, not only image counts.
L3 Routine variation	CLAIM and STARD-AI: data handling, reference standard, and applicability [57,58]	Challenge sanitation, line speed, presentation, natural defects, and non-target materials. Healthcare checklists do not prescribe food challenge levels.
L4 Named external shifts	TRIPOD + AI, CLAIM, and STARD-AI: evaluation populations and generalizability [56,57,58]	Separate time, instrument, supplier, and site; prespecify margins, sample size, and acceptance for each claimed shift.
L5 Prospective action and outcomes	Reporting principles alone do not establish intervention benefit	Test item identity, actuation, accepted-product outcomes, and any feedback intervention. Codex hazard-control requirements remain separate [52].

Note: L1–L5 identify partly independent evidence dimensions; they do not denote cumulative levels, maturity gradations, or readiness scores.

**Table 5:** Proposed checks for acquisition-to-actuation validation. Installation-specific limits must be justified before testing.

Validation Item	Challenge and Recorded Evidence	Prespecified Acceptance or Response
Coverage and synchronization	Known items through all views; encoder/timestamp alignment; occlusion, glare, overlap, variable speed and spacing; count all presented items	Required coverage and allowable timing/position error; uninspectable items held or routed for validated reinspection
End-to-end latency	Exposure/scan to decision and actuation; tail latency, jitter, peak load, communication delay and dropped frames	Deadline derived from speed, distance, spacing and actuator response; deadline breach activates defined fallback
Item identity and spatial alignment	Track known positive and negative items; test slippage, belt stops, restarts and close neighbors	Limits on identity loss and wrong-item rejection; unresolved identities included in system failures
Physical rejection	Challenge target materials, sizes and positions; record commands, ejections, bounce-back and incomplete removal	Correct removal confirmed downstream; report missed rejection and false rejection with explicit denominators
Independent downstream confirmation	Blind checks of accepted and rejected streams using endpoint-appropriate references across batches/shifts	Residual defect/hazard and false-reject bounds meet the intended-use criteria; hazard-control failures trigger hold and investigation; quality-only deviations trigger review and corrective action.
Faults and recovery	Camera/lighting/encoder failure; network/power loss; blocked ejector; overload; test recovery and restart	Safety-related failures trigger fail-safe hold/stop or validated fallback; quality/throughput-only failures trigger maintenance or review. Recovery requires defined checks and, where applicable, disposition of affected product.
Prospective control and change	Locked versions; baseline and intervention periods; transport delay; overshoot; overrides; outcome and change logs	Stability and endpoint criteria met before use; safety limits maintained; material updates require independent revalidation

Failure-response classification: Criticality depends on intended use and hazard analysis. Safety-critical failures include inadequate coverage or synchronization, missed actuation deadlines, loss of item identity or spatial alignment, or failed removal when these compromise a required hazard control; downstream hazard-limit failures; faults that disable that control; and safety-limit violations or unvalidated changes to a safety-related configuration. Hold affected product and stop the affected operation when continued safe control cannot be assured, unless a validated fallback maintains control. Apply defined corrective-action and release/restart criteria. Failures confined to quality or throughput, such as excess false rejection, reduced capacity, or quality-target drift while validated safety controls remain effective, require review, maintenance, or adjustment/revalidation as appropriate, without an automatic safety hold/stop. Any uncertainty about safety-control integrity follows the safety-critical response. Prespecify the response and responsible decision-maker for each item [52].

**Table 6:** Inspection failure modes, controls, and triggers for investigation or revalidation.

Failure Mode	Observable Signal	Preventive or Verification Control	Escalation Trigger
Optical drift	Blur, glare, radiometric change, or failed reference	Fixed acquisition; cleanliness checks; reference targets	Acquisition check outside its acceptance limit
Product or domain change	Subgroup errors or changing reference residuals	Representative accepted-product checks; subgroup and drift monitoring	Confirmed performance below the approved criterion
Reference or data error	Label disagreement, bias, or duplicated sample lineage	Reference quality control; traceable samples; grouped partitions	Invalid labels or compromised evaluation independence
Prevalence or threshold mismatch	Excess review, false rejection, or escapes	Track denominators, prevalence, threshold version, and outcomes	Decision burden or hazard-control criterion exceeded
Software, model, or sensor change	Changed outputs after an update or replacement	Versioned configuration; transfer checks; independent revalidation	Unexplained output change or failed acceptance test
Unknown defect or confounder	Novel material, sensor fault, or high-confidence error	Defined hold route; adjudication; new challenge examples	Serious escape or recurring confirmed new failure
Acquisition–actuation mismatch	Late decision, lost identity, or incomplete ejection	Synchronized tracking and downstream removal verification	Wrong-item rejection or timing outside validated bounds

An alert initiates diagnosis and the defined product-control response; it does not automatically justify retraining. Maintenance, reference correction, threshold review, and model revalidation address different causes. Specific limits and responsibilities depend on the intended use and the established food-control system.

## Data Availability

No new data were created or analyzed in this study. Data sharing is not applicable to this article.

## Supplementary Materials

The following supporting information can be downloaded at: https://www.mdpi.com/article/10.3390/foods15203591/s1. Table S1. Structured qualitative appraisal of the 13 empirical examples in Table 2, with source-specific sample, reference, grouping, environment, and interpretation fields.
