# Supplementary material for: Artificial Intelligence-Enabled Computer Vision for Food Inspection: From Validated Measurements to Process Control
Source: Foods. 2026 Oct 10;15(20):3591. doi: 10.3390/foods15203591 (PMC13704548; doi:10.3390/foods15203591)
Supplement: Supplementary file 1 [file foods-15-03591-s001.zip › foods-4598603-supplementary.pdf]

Supplementary

Table S1. Structured qualitative appraisal of the 13 selected empirical examples in Table 2.

Study and endpoint	Samples and derived observations	Reference and data grouping	Environment and evidence dimension	Qualitative limitations
Apple/banana appearance [24]	Reported image datasets; live trial: 30 apples and 32 bananas	Appearance labels; 80:10:10 image split; physical-item grouping unresolved	Lighting-box prototype; internal plus live-camera testing	Small live set; single view; no conveyor validation; inconsistent reported offline image totals
Avocado ripeness [26]	80 fruit; 551 longitudinal images; 44,096 reported patches	Firmness < 15 N defines ripeness; patches from each image cross partitions	Laboratory HSI; internal patch prediction	Correlated patches/repeated fruit; mean per-image RMSE of 1.17 days is not independent-fruit error; reported patch totals do not reconcile with the stated patches per image
Apple composition [30]	96 apples in 2023; 48 in 2024	DCPIP, refractometry, Bradford; 2023 split 7:3; 2024 holdout	Laboratory HSI; temporal external validation	Small holdout; shared cultivar/origin groups; independent instrument or imaging-site transfer not reported
Maize AFB1 categories [33]	450 kernels: 225 calibration and 225 prediction; 150 newly prepared validation kernels	Nominal toxin dosing; random kernel split; new preparation	Laboratory NIR-HSI; prepared-sample challenge	Artificial dose groups; natural contamination and lot exposure unresolved
Walnut internal quality [10]	More than 8000 nuts; 1756 images; three online output collections of at least 500 nuts each	Cut-open quality grades; 7:2:1 image split; nut grouping unresolved	Conveyor X-ray/pneumatic sorting; output inspection	Resealed training nuts; short trial; reported ejection precision is accepted-stream purity
Tea foreign objects [34]	120 subsamples; 836 retained images → 9,196 augmented	Manual boxes; 920 test images split after augmentation; 10-fold CV	External scenes and Raspberry Pi conveyor station	Augmentation-family leakage risk; external sample denominators unresolved; no removal endpoint
Trout TVB-N [38]	40 fish → 210 subsamples; 140/70 calibration/prediction	Micro-Kjeldahl distillation; parent-fish grouping unresolved	Laboratory cold storage; within-study prediction	One source/storage regime; correlated subsamples; not pathogen detection
Cod nematodes [39]	335 images; 289 retained/244 nematodes; 196/49/44 split	Expert candling/polygon masks; random sample split	One factory/three dates; industrial imaging/inference	46 difficult images excluded; imperfect reference; no removal outcome
Poultry foreign objects [40]	12 fillets/96 images; 6 large-object development/6 small-object test	Known placed objects/manual ROIs; fillet-level size challenge	Laboratory VNIR/SWIR; controlled detection	Transparent/semitransparent objects excluded; surface placements; small test

Study and endpoint	Samples and derived observations	Reference and data grouping	Environment and evidence dimension	Qualitative limitations
Poultry model acceleration [42]	12 fillets/48 reused images	Known object labels; all images reused, including earlier training set	Archived HSI benchmarking; throughput simulation	Computational fidelity/speed only; no fresh independent industrial rejection trial
Thermoformed seals [44]	3606 development images; 6576 normal/4304 disturbed-line products	Visual criteria/manual checks; 2978/628 image split	Commercial line; 150/105 min production challenges	Image grouping unresolved; visual defects ≠ leaks; 206/209 sensitivity = 98.56%
Pizza closures [43]	4558 seal crops; 3647/455/456 split; package n unresolved	Visual criteria/manual supervision; image split; best of 5 runs	Industrial conveyor; three brands/two toppings	Best-run reporting; crop dependence; no functional integrity reference
Cultured bacteria/debris [45]	Training: 3 days; 315 images per strain; 210 per matrix. GFP challenge: 2 days; 60 images; 133 colonies	Manual labels/GFP confirmation; 60:10:30 images; day grouping unresolved	Laboratory imaging after 3 h incubation; preparation and imaging add time; three matrices	Object-level result; not 133 independent food samples; no official-method equivalence

Abbreviations: AFB1, aflatoxin B1; DCPIP, 2,6-dichlorophenolindophenol; GFP, green fluorescent protein; HSI, hyperspectral imaging; NIR, near-infrared; ROI, region of interest; RMSE, root mean square error; SWIR, short-wave infrared; TVB-N, total volatile basic nitrogen; VNIR, visible–near-infrared.

Source locations and interpretation notes

[24] Ismail, N., & Malik, O. A. (2022). Real-time visual inspection system for grading fruits using computer vision and deep learning techniques. Information Processing in Agriculture, 9(1), 24–37. https://doi.org/10.1016/j.inpa.2021.01.005. Source locations: Sections 2.3.1 and 2.3.3; live-sample evaluations and conclusions. Reported offline image totals are internally inconsistent, so no independent sample size is inferred from them.

[26] Davur, Y. J., Kämper, W., Khoshelham, K., Trueman, S. J., & Bai, S. H. (2023). Estimating the ripeness of Hass avocado fruit using deep learning with hyperspectral imaging. Horticulturae, 9(5), 599. https://doi.org/10.3390/horticulturae9050599. Source locations: Sections 2.1–2.4 and Table 1. Patches from each image entered all partitions; patch totals do not reconcile with the stated patches per image.

[30] Zhai, H., Xie, P., Xie, X., & Sha, S. S. (2025). Deep learning-enabled hyperspectral imaging for high-accuracy non-destructive quantification of nutritional components in multi-variety apples. Frontiers in Plant Science, 16, 1634785. https://doi.org/10.3389/fpls.2025.1634785. Source locations: Sections 2.2–2.4 and 3.4.

[33] Zhou, Q., Huang, W., Liang, D., & Tian, X. (2021). Classification of aflatoxin B1 concentration of single maize kernel based on near-infrared hyperspectral imaging and feature selection. Sensors, 21(13), 4257. https://doi.org/10.3390/s21134257. Source locations: Sections 2.1, 2.5 and 3.7; Table 5.

[10] Fan, X., & Zhou, J. (2025). Nondestructive detection and quality grading system of walnut using X-ray imaging and lightweight WKNet. Foods, 14(13), 2346. https://doi.org/10.3390/foods14132346. Source locations: Sections 2.1.2–2.1.3, 3.1 and 3.3; Table 5.

[34] Wang, Z., Wang, C., Yuan, W., Deng, X., Wang, H., Wu, T., Zhao, J., Liu, W., & Wang, B. (2026). Non-destructive detection of micro-impurities in tea using the YOLOv11-PFT model. npj Science of Food, 10(1), 52. https://doi.org/10.1038/s41538-025-00702-6. Source locations: Methods on image preprocessing and dataset division; external validation and edge-deployment results; Tables 4 and 6.

[38] Moosavi-Nasab, M., Khoshnoudi-Nia, S., Azimifar, Z., & Kamyab, S. (2021). Evaluation of the total volatile basic nitrogen (TVB-N) content in fish fillets using hyperspectral imaging coupled with deep learning neural network and meta-analysis. Scientific Reports, 11(1), 5094. https://doi.org/10.1038/s41598-021-84659-y. Source locations: Methods on samples, reference TVB-N determination and model calibration; prediction results.

[39] Syed, S., Ortega, S., Anderssen, K. E., Nilsen, H. A., & Heia, K. (2024). Hyperspectral imaging and deep learning for parasite detection in white fish under industrial conditions. Scientific Reports, 14(1), 27426. https://doi.org/10.1038/s41598-024-76808-w. Source locations: Methods on industrial acquisition, reference annotation and dataset selection; detection results.

[40] Chung, S., & Yoon, S.-C. (2021). Detection of foreign materials on broiler breast meat using a fusion of visible near-infrared and short-wave infrared hyperspectral imaging. Applied Sciences, 11(24), 11987. https://doi.org/10.3390/app112411987. Source locations: Methods on placed materials, fillet allocation and spectral fusion; material- and size-specific results.

[42] Khan, Z., Yoon, S.-C., & Bhandarkar, S. M. (2025). Deep learning model compression and hardware acceleration for high-performance foreign material detection on poultry meat using NIR hyperspectral imaging. Sensors, 25(3), 970. https://doi.org/10.3390/s25030970. Source locations: Methods on the reused HSI dataset, compression and camera assumptions; benchmarking and simulated throughput results.

[44] Banús, N., Boada, I., Xiberta, P., Toldrà, P., & Bustins, N. (2021b). Deep learning for the quality control of thermoforming food packages. Scientific Reports, 11(1), 21887. https://doi.org/10.1038/s41598-021-01254-x. Source locations: Tables 1, 4 and 9; final production-test results.

[43] Banús, N., Boada, I., Bardera, A., & Toldrà, P. (2021a). A deep-learning based solution to automatically control closure and seal of pizza packages. IEEE Access, 9, 167267–167281. https://doi.org/10.1109/access.2021.3135973. Source locations: Sections IV–V and Tables 3–8; laboratory and industrial evaluation.

[45] Park, H. W., Li, Z., Ma, L., & Nitin, N. (2025). Deep learning enabled rapid detection of live bacteria in the presence of food debris. npj Science of Food, 9(1), 274. https://doi.org/10.1038/s41538-025-00636-z. Source locations: Methods on images and model training; GFP-producing B. subtilis food-matrix validation results. The three-day training dataset and two-day GFP validation are distinct.
